# Supplementary material for: Maternity care providers’ perspectives on late-term gestation management (LATE-study): A cross-sectional survey
Source: PLoS One. 2025 Aug 18;20(8):e0329843. doi: 10.1371/journal.pone.0329843 (PMC12360596; doi:10.1371/journal.pone.0329843)
Supplement: S1 File — (DOCX) [file pone.0329843.s001.docx]

**Supporting information: Questionnaire LATE-study**

## Introduction

This scientific study focuses on the policy at 41 weeks of pregnancy, as described in the multidisciplinary guideline 'Policy Pregnancy from 41 Weeks’ ([Beleid zwangerschap vanaf 41 weken - Richtlijn - Richtlijnendatabase](https://richtlijnendatabase.nl/richtlijn/beleid_zwangerschap_41_weken/beleid_zwangerschap_vanaf_41_weken.html)). We are curious about your opinion on this policy and the use of this guideline. The questionnaire will take approximately 20-30 minutes of your time.

## Consent

**Researchers**: This study is conducted by the departments of Midwifery Science and Obstetrics & Gynecology at Amsterdam UMC.

**Objective of the Study**: The purpose of this questionnaire is to investigate the opinions of healthcare providers about the guideline and its practical application. Your participation contributes to a better understanding of this subject.

**Voluntary Participation**: Participation in this study is voluntary. You may decide to stop during the questionnaire, and you are not required to provide a reason. Of course, we greatly appreciate it if you complete the entire questionnaire.

**Use of Your Data**: If you participate in the study, you Ifo give consent to collect, use, and store your data. The results of the study will be published and may be used as input for potential improvements to the guideline or its implementation. Participation is entirely anonymous. We will keep your data for a maximum of 10 years in the research department and may Ifo use the data for follow-up research. For more information about your rights regarding the processing of your personal data, you can contact the researchers of this study. If you are dissatisfied with how your privacy is handled, you can file a complaint with the Data Protection Officer at … (e-mail address). You can Ifo contact the Dutch Data Protection Authority via …..

**Questions?** This study has been reviewed by the ethics committee of Amsterdam UMC. According to this committee, this study does not fall under the Dutch Medical Research Involving Human Subjects Act (WMO). If you have questions about this study, please contact the researchers.

**Contact Information**: … (e-mail addresses of researchers)

| Consent | Are you in agreement with the statements below? | Yes  No |
| --- | --- | --- |

1. I have read the information about this study.
2. I understand that participation is voluntary. I understand that I can decide at any time not to participate or to stop the study. I do not have to explain the reasons behind this.
3. I consent to the collection and use of my data in the manner and for the purposes stated in the previous information.
4. I give permission to keep my data for a maximum of 15 years after this research within ….
5. I want to participate in this research.

| Information | |
| --- | --- |
| *Note that “Induction of labour” in this survey is defined as an artificially onset of labour through cervical ripening, amniotomy and/or oxytocin, before the membranes rupture spontaneously or contractions start spontaneously.*  *---------------------------------*  The guideline consists of two modules, each with two recommendations. In this questionnaire, we primarily focus on module 1 ('Policy Pregnancy from 41 Weeks'). Some questions will pertain to module 2 ('Antepartum Fetal Monitoring from 41 Weeks in Expectant Management'). In such cases, we will make it clear in the question. You can find the guideline using the following link: [Beleid zwangerschap vanaf 41 weken - Richtlijn - Richtlijnendatabase](https://richtlijnendatabase.nl/richtlijn/beleid_zwangerschap_41_weken/beleid_zwangerschap_vanaf_41_weken.html) | |
| What is your main profession? | 1 = Obstetrician 2 = Obstetric registrar (AIOS) 3 = Obstetric registrar (ANIOS) 4 = Midwife clinically working (PA specialized) 5 = Midwife clinically working (not PA specialized) 6 = Midwife primary care 7 = General practitioner 8 = Physician specialized in obstetrics 9 = Other |
| Are you still practising within maternity care? | 1 = Yes 0 = No |
| If No:  When did you stop practising? | 1 = Less than 6 months ago 2 = 6 months – 1 year ago 3 = More than 1 year ago |
| How often do you perform counselling on late-term pregnancies? | 0 = Never 1 = Less than once a month 2 = Once a month 3 = Once in two weeks 4 = Every week or more often |
| **Awareness** | |
| *Module 1:*  *The two recommendations in module 1 are as follows:*  *1.1) Offer the pregnant woman with a singleton in cephalic presentation the option of inducing labor from 41 weeks (287 days amenorrhea) alongside the option to wait.*  *1.2) Counsel using the choice cards* associated with the module and specify the effects on perinatal outcomes such as perinatal mortality and NICU admissions, and the potential increased risk for nulliparous compared to multiparous women. In this context, the advantages and disadvantages of both induction and expectant management should be discussed.*  **Choice Cards:* [*Inleiden of afwachten*](https://www.thuisarts.nl/file/3110/download?token=RJJxDvg_) *and* [*Andere mogelijkheden*](https://www.thuisarts.nl/file/3112/download?token=iC6ykImL) | |
| Was you aware of the existence of module 1 of the guideline? | 1 = Yes 0 = No |
| Have you read module 1 of the guideline? | 1 = Yes, only the recommendations 2 = Yes, I have read further than the recommendations once 3 = Yes, I have read further than the recommendations more than once 0 = No, never |
| *Module 2:*  *The two recommendations in module 2 are as follows:*  *2.1) Discuss with the pregnant woman the limitations of fetal monitoring at 41 weeks if she wishes to wait.*  *2.2) Specify that the cardiotocography (CTG) and ultrasound, in the context of expectant management beyond 41 weeks, do not have a good predictive value for pregnancy outcomes.* | |
| Was you aware of the existence of module 2 of the guideline? | 1 = Yes 0 = No |
| Have you read module 2 of the guideline? | 1 = Yes, only the recommendations 2 = Yes, I have read further than the recommendations once 3 = Yes, I have read further than the recommendations more than once 0 = No, never |
| **Opinion and application** | |
| *Module 1:*  *The first recommendation of module 1 is as follows:*  *1.1) Offer the pregnant woman with a singleton in cephalic presentation the option of inducing labor from 41 weeks (287 days amenorrhea) alongside the option to wait.* | |
| Do you agree with this recommendation? | Standard Likert scale: 1 = Strongly disagree 2 = Disagree 3 = Neither agree nor disagree 4 = Agree 5 = Strongly agree |
| If disagree:  To which of the following policy option do you agree with? | Multiple choice:  1 = All women should be adviced to be induced at 41+0 weeks of gestation. 2 = The choice to be induced should be offered at another time point than at 41+0. 3 = Expectant management until 42+0 weeks should be standard management for low-risk women. 4 = Expectant management without boundary should be standard management for low-risk women. 5 = The choice to be induced at 41+0 weeks should only be offered to nulliparous women. 6 = The choice to be induced at 41+0 weeks should be offered to all women, but nulliparous women should be adviced to be induced. 7 = Other, namely (see next question): |
| If ‘The choice to be induced should be offered at another time point than at 41+0.’:  At which time point induction of labour should be offered in your opinion? | Open-ended |
| If ‘Other, namely:  Other, namely’: | Open-ended |
| Do you apply this recommendation in daily practice? | Frequency Likert scale:  1 = Never 2 = Rarely 3 = Sometimes 4 = Often 5 = Always |
| *We split the second recommendation in four parts, in order to get more insight into your perspectives on the different parts of the recommendation*  *1.2a) Counsel using the choice cards* associated with the module.* | |
| Do you agree with this recommendation (that counselling should take place using the choice cards)? On the next page, questions will follow about the content of the choice cards. | Standard Likert scale |
| Do you apply this recommendation in daily practice? | Frequency Likert scale |
| *1.2b) Specify the effects on perinatal outcomes such as perinatal mortality and NICU admissions.* | |
| Do you agree with this recommendation? | Standard Likert scale |
| Do you apply this recommendation in daily practice? | Frequency Likert scale |
| *1.2c) Specify the potential increased risk for nulliparous compared to multiparous women.* | |
| Do you agree with this recommendation? | Standard Likert scale |
| Do you apply this recommendation in daily practice? | Frequency Likert scale |
| *1.2d) In this context, the advantages and disadvantages of both induction and expectant management should be discussed.* | |
| Do you agree with this recommendation? | Standard Likert scale |
| Do you apply this recommendation in daily practice? | Frequency Likert scale |
| *Module 2:*  *The first recommendation of module 2 is as follows:*  *2.1)* *Discuss with the pregnant woman the limitations of fetal monitoring at 41 weeks if she wishes to wait.* | |
| Do you agree with this recommendation? | Standard Likert scale |
| Do you apply this recommendation in daily practice? | Frequency Likert scale + Not applicable, since I do not encounter this situation in my work |
| *Module 2:*  *The second recommendation of module 2 is as follows:*  *2.2)* *Specify that the cardiotocography (CTG) and ultrasound, in the context of expectant management beyond 41 weeks, do not have a good predictive value for pregnancy outcomes.* | |
| Do you agree with this recommendation? | Standard Likert scale |
| Do you apply this recommendation in daily practice? | Frequency Likert scale + Not applicable, since I do not encounter this situation in my work |
| Optional: clarify one or more of the answers above. | Open-ended |
| Do you miss elements in the guideline? | 0 = No 1 = Yes, namely (see next question): |
| Yes, namely: | Open-ended |
| Do you find elements in the guideline should be changed? | Yes  No  Unsure |
| If Yes:  To which of the following elements changes should be made in your opinion? | Multiple choice:  1 = Inclusion of all pregnant women >41 weeks 2 = Included studies 3 = Included outcome measures 4 = Described considerations 5 = Deviation in characteristics (with regard to the recommendations) 6 = Content of the counselling 7 = Other, namely (see next question): |
| Other, namely: | Open-ended |
| If agree with ‘Inclusion of all pregnant women >41 weeks’:  What should be changed with regard to the inclusion of all pregnant women >41 weeks? | Open-ended  Optional |
| If agree with ‘Included studies’:  What should be changed with regard to the included studies? | Open-ended  Optional |
| If agree with ‘Included outcome measures’:  What should be changed with regard to the outcome measures? | Open-ended  Optional |
| If agree with ‘Described considerations’:  What should be changed with regard to the considerations? | Open-ended  Optional |
| If agree with ‘Deviation in characteristics (with regard to the recommendations)’:  Wat should be changed with regard to the deviation in characteristics (with regard to the recommendations)? | Open-ended  Optional |
| If agree with ‘Content of the counselling’:  What should be changed with regard to the content of the counselling? | Open-ended  Optional |
| I have confidence in the working group who developed the guideline. | Standard Likert Scale + Unsure |
| Optional: clarify one or more of the answers above. | Open-ended |
| **Counselling** | |
| *The guideline includes three tools (see link):*  *1) Counselling using BRAINS-method*  *Link:* [*Beleid zwangerschap vanaf 41 weken - Richtlijn - Richtlijnendatabase*](https://richtlijnendatabase.nl/richtlijn/beleid_zwangerschap_41_weken/beleid_zwangerschap_vanaf_41_weken.html#attachment) | |
| Was you aware of the existence of this tool? | 1 = Yes 0 = No |
| This tool useful and helpful in my opinion. | Standard Likert scale |
| If disagree:  Why do you find this tool not useful/helpful? | Open-ended |
| *2) Choice card 1 (‘Induction or expectant management’)*  Link: <https://www.thuisarts.nl/file/3110/download?token=RJJxDvg_> | |
| Was you aware of the existence of this tool? | 1 = Yes 0 = No |
| This tool useful and helpful in my opinion. | Standard Likert scale |
| If disagree:  Why do you find this tool not useful/helpful? | Open-ended |
| *3) Choice card 2 (‘Other choice options)*  Link: <https://www.thuisarts.nl/file/3112/download?token=iC6ykImL> | |
| Was you aware of the existence of this tool? | 1 = Yes 0 = No |
| This tool useful and helpful in my opinion. | Standard Likert scale |
| If disagree:  Why do you find this tool not useful/helpful? | Open-ended |
| I (also) use other tools for counselling. | 0 = No 1 = Yes, namely |
| Yes, namely: | Open-ended |
| Ruimte voor eventuele toelichting op jouw antwoorden op bovenstaande vragen. | Open-ended |
| *The following questions concern counseling conversations on policy in late-term pregnancies. Mark the topics that are part of 75% of the counselling conversation.* | |
| Mark the topics that are part of 75% of the counselling conversation. | 1 = Percentages of outcomes for each decision option 2 = Advantages of induction of labour 3 = Advantages of expectant management 4 = Disadvantages of induction of labour 5 = Disadvantages of expectant management 6 = Differences in outcomes between nulliparous and multiparous women  7 = Alternatives: stripping of the membranes 8 = Alternatives: artificially rupturing the membranes prior to the onset of labour 9 = Alternatives: expectant management beyond 42 weeks 10 = None of the above mentioned |
| Mark the issues that are part of 75% of the counselling conversation. | 1 = Asking whether counselling is desired 2 = Recognising preferences of the pregnant woman (and her partner) 3 = Whether the pregnant woman (and her partner) understood the information 4 = Not yet making a decision is an option (labour may start spontaneously in the meantime) 5 = Giving time to think 6 = Invite to ask questions  7 = None of the above mentioned |
| Nulliparous women: I tend to emphasize the advantages of induction of labour above the advantages of expectant management. | Frequency Likert scale |
| Multiparous women: I tend to emphasize the advantages of induction of labour above the advantages of expectant management. | Frequency Likert scale |
| Nulliparous women: I tend to emphasize the advantages of expectant management above the advantages of induction of labour | Frequency Likert scale |
| Multiparous women: I tend to emphasize the advantages of expectant management above the advantages of induction of labour. | Frequency Likert scale |
| The preference of the woman is the most important topic of discussion. | Frequency Likert scale |
| The preference of the woman is the most important factor in the decision making. | Frequency Likert scale |
| Pregnant women follow my advice, because they trust my expertise. | Frequency Likert scale |
| Pregnant women have difficulties to translate the information into a decision that fits their personal values. | Frequency Likert scale |
| In case of a language barrier or functional illiteracy, I do not perform the whole counselling. | Frequency Likert scale |
| I have difficulties with mentioning the chance that the baby dies. | Frequency Likert scale |
| Optional: clarify one or more of the answers above. | Open-ended |
| **Impact practice** | |
| *Give your opinion on the following statements:*  *Following the recommendations in the guideline results in my practice/hospital in:* | |
| improved outcomes for women at 41 weeks of gestation and beyond. | Standard Likert scale + Unsure |
| improved outcomes for babies at 41 weeks of gestation and beyond. | Standard Likert scale + Unsure |
| increased client-centred care. | Standard Likert scale + Unsure |
| improvement for the organisation of maternity care for women at 41 weeks of gestation and beyond. | Standard Likert scale + Unsure |
| changes on care for other women not yet being 41 weeks of gestation. | Standard Likert scale + Unsure |
| improvement of care for other women not yet being 41 weeks of gestation. | Standard Likert scale + Unsure |
| deterioration of care for other women not yet being 41 weeks of gestation. | Standard Likert scale + Unsure |
| increased fear and/or less confidence in a normal process of pregnancy in women. | Standard Likert scale + Unsure |
| increased medicalisation. | Standard Likert scale + Unsure |
| (more) problems with the capacity of maternity care. | Standard Likert scale + Unsure |
| Optional: clarify one or more of the answers above. | Open-ended |
| Offering induction at 41 weeks to all women and inducing women at 41+0 who prefer this, is possible in my region of work. | Frequency Likert scale + Unsure |
| *Module 1:*  *The first recommendation of module 1 is as follows:*  *1.1) Offer the pregnant woman with a singleton in cephalic presentation the option of inducing labor from 41 weeks (287 days amenorrhea) alongside the option to wait.* | |
| Is this recommendation applied in your working region? | Yes  No  Unsure |
| *There are different periods in which obstetric care providers started offering inductions from 41 weeks:*  *1) After the publication of the national guideline in the Netherlands.*  *2) Even before the publication of the guideline but after the publication of the INDEX or SWEPIS studies in 2019: studies comparing induction at 41 weeks with expectant management until 42 weeks at the latest.*  *3) Even earlier, namely after the publication of the Dutch Society of Obstetrics and Gynaecology (NVOG) guideline on Prolonged Pregnancy in 2007. It recommends that if parents request induction between 41 and 42 weeks without apparent risk factors, the pregnancy can be terminated by inducing labor after providing information about induction.* | |
| Since when has this policy been applied in your working region? | 1 = After release of the national guideline (from 2021 onward) 2 = After the INDEX-study (2019), but before release of the guideline (2021) 3 = After an earlier guideline of obstetricians (2007), but before the INDEX-study (2019) 4 = Other, namely (see next question) 9 = Unsure |
| Other, namely: | Open-ended |
| *If 2nd or 3rd choice: Fill in the following questions for the period between 2019 and 2021 / 2007 and 2019.* | |
| Are adjustments made in your hospital in order to apply this recommendation? | Yes  No  Unsure |
| If Yes:  Which adjustments are made? | Open-ended |
| If primary care:  Are adjustments made in the hospital you work with in order to apply this recommendation? | Yes  No  Unsure |
| If Yes:  Which adjustments are made? | Open-ended |
| If primary care:  Are adjustments made in the primary care practice you work most frequently, in order to apply this recommendation? | Yes  No  Unsure |
| If Yes:  Which adjustments are made? | Open-ended |
| My work changed significantly due to the application of the recommendations in guideline. | Standard Likert scale + Unsure |
| If agree:  I experience these changes positive. | Standard Likert scale |
| How does the recommendations in this guideline impact your work or you as a care provider? | 0 = Not at all 1 = As follows: |
| As follows: | Open-ended |
| Optional: clarify one or more of the answers above. | Open-ended |
| **Characteristics** | |
| What is your gender? | 1 = Male 2 = Female 3 = Prefer not to answer |
| What is your age? | Min 20, max 80 |
| What is the total number of years with working experience in maternity care? | Min 0, max 50 |
| What is your (main) country of education as a maternity care professional? | 0 = The Netherlands 1 = Abroad, namely (see next question): |
| Abroad, namely | Open-ended |
| If midwife:  What is your highest level of education? | 0 = Bachelor degree 1 = Master degree 2 = PhD |
| What form of employment do you work in most frequently? | 1 = Paid employment 2 = Self-employed without partnership 3 = Partnership 4 = Other, namely (see next question): |
| Other, namely: | Open-ended |
| If obstetrician:  What is your main sub-speciality? | 1 = Obstetrian/perinatology 2 = Urogynaecology 3 = Reproductive medicine 4 = Oncology 5 = Benign gynaecology 6 = General gynaecologist |
| If obstetrician:  How common do you work at an obstetric unit? | 0 = Never 1 = Less than monthly 2 = Monthly 3 = Two-weekly 4 = Weekly |
| In which region do you work most frequently? | List of regions  Other, namely: |
| Other, namely: |  |
| If secondary care:  In which type of hospital do you work most frequently? | 1 = Tertiary/academic center 2 = Secondary/peripheral center |
| If secondary care:  How many births take place yearly in the hospital you work most frequently? | 0 = <500 1 = 500-1000 2 = 1000-1500 3 = 1500-2000 4 = >2000 5 = Unsure |
| If midwife primary care:  What is the practice size of the practice where you work most frequently? | 0 = 1-2 midwives 1 = 3-5 midwives 2 = 6-10 midwives 3 = 11 or more midwives |
| Do you experience a problem with the capacity in terms of staff and maternity beds in your region of work? | Frequency Likert scale + Unsure |
| If primary care:  Of 10 births that require transfer from home to hospital (with or without referral to secondary care): How often can you transfer to the hospital of first choice? | 0-10 |
| National guidelines (in general) optimize health care delivery and outcomes by supporting patient-clinician communication and decision-making. | Standard Likert scale + Unsure |
| I possess sufficient knowledge about the scientific rationale and foundation of the guideline in order to assess its quality. | Standard Likert scale + Unsure |
| **Birth Beliefs Scale** | |
| Birth is a medical event. | Standard Likert scale for each statement. |
| Nowadays, there is no reason why women should suffer pain in childbirth. |  |
| A woman’s body knows how to give birth. |  |
| There are many things that can go wrong during childbirth. |  |
| Labour should be allowed to proceed at its own pace. |  |
| Often, a woman’s body structure does not allow her to give birth naturally. |  |
| Birth is a natural event. |  |
| Pain in childbirth is a significant part of the birth experience. |  |
| Childbirth requires vigilant medical supervision. |  |
| Childbirth is a dangerous process. |  |
| Birth is an empowering experience. |  |

Thank you very much for your participation in this questionnaire.

You have made a significant contribution to scientific research with the aim of gaining more insight into the perspectives of healthcare providers regarding management policies for pregnancies from 41 weeks onwards.
